# Structural Characterization of Mucin O-Glycosylation May Provide Important Information to Help Prevent Colorectal Tumor Recurrence

**DOI:** 10.3389/fonc.2015.00217

**Published:** 2015-10-08

**Authors:** Adriana Mihalache, Jean-François Delplanque, Bélinda Ringot-Destrez, Cindy Wavelet, Pierre Gosset, Bertrand Nunes, Sophie Groux-Degroote, Renaud Léonard, Catherine Robbe-Masselot

**Affiliations:** ^1^Structural and Functional Glycobiology Unit, UMR CNRS 8576, University of Lille, Villeneuve d’Ascq, France; ^2^Service d’Anatomie Pathologie, Faculté Libre de Médecine, Hôpital Saint Vincent de Paul, Groupement des Hôpitaux de l’Institut Catholique de Lille, Lille, France; ^3^Service Chirurgie Digestive, Faculté Libre de Médecine, Hôpital Saint Philibert, Groupement des Hôpitaux de l’Institut Catholique de Lille, Lille, France

**Keywords:** colorectal cancer, recurrence, glycosylation, human intestinal mucins, prognosis

## Abstract

Although colorectal cancer is a preventable and curable disease if early stage tumors are removed, it still represents the second cause of cancer-related death worldwide. Surgical resection is the only curative treatment but once operated the patient is either subjected to adjuvant chemotherapy or not, depending on the invasiveness of the cancer and risks of recurrence. In this context, we investigated, by mass spectrometry (MS), alterations in the repertoire of glycosylation of mucins from colorectal tumors of various stages, grades, and recurrence status. Tumors were also compared with their counterparts in resection margins from the same patients and with healthy controls. The obtained data showed an important decrease in the level of expression of sialylated core 3-based O-glycans in tumors correlated with an increase in sialylated core 1 structures. No correlation was established between stages of the tumor samples and mucin O-glycosylation. However, with the notable exception of sialyl Tn antigens, tumors with recurrence presented a milder alteration of glycosylation profile than tumors without recurrence. These results suggest that mucin O-glycans from tumors with recurrence might mimic a healthier physiological situation, hence deceiving the immune defense system.

## Introduction

Colorectal cancer (CRC) is the second leading cause of cancer-related death in the Western world (1). It arises as the result of an accumulation of genetic and epigenetic alterations leading to transformation of normal colonic epithelium to invasive adenocarcinomas, due to disorders in cell proliferation and differentiation (2).

Unlike many other malignancies, CRC is a preventable and curable disease if early stage tumors are removed. Actually surgical resection complemented or not with adjuvant chemotherapy is the only curative treatment. However, patient survival is highly dependent on the tumor stage at the time of diagnosis. Metastatic CRC is frequently associated with recurrences within 5 years after surgery. In this context, there is an urgent need to identify biomarkers for early detection of CRC, to follow tumor development during the course of therapy and to predict recurrence.

Screening of risk populations currently remains based on fecal occult blood test and on colonoscopy for the possible discovery of polyps and invasive tumors. Despite the accuracy of these diagnostic methods, many CRC remain undetected until they have spread to the surrounding organs or lymph, correlating with poor prognosis because of the low sensitivity and specificity of these tests (3). Knowledge of the molecular mechanisms of colorectal carcinogenesis will help to improve tumor detection as well as treatment.

Altered glycosylation is a universal hallmark of cancer (4, 5). Many tumor-associated antigens defined by monoclonal antibodies raised against cancer cells are cell-surface carbohydrate epitopes (6, 7). Among all glycoproteins, mucins, the major component of the mucus gel covering and protecting the intestinal tract, are frequently altered in colon cancers. Alterations of mucin O-glycosylation include expression of relatively short truncated O-glycans and altered peripheral structures (8–13). Three types of alterations can lead to tumor-associated O-glycans: (i) neoexpression of antigens that are not expressed in embryonic or healthy tissues; (ii) expression of oncofetal antigens that are absent or rare in healthy adult tissues but expressed during embryonic development; and (iii) modified expression levels of antigens found in healthy tissues. All these alterations are important in tumor biology and can be useful in clinical management (14). In CRC, alterations of mucin O-glycans include reduced expression of core 3 (GlcNAcβ1-3GalNAcα-Ser/Thr) and core 4 [GlcNAcβ1-3(GlcNAcβ1-6)GalNAcα-Ser/Thr] based glycans (15, 16). Other features are an increased sialylation of mucin O-glycans and a decreased sulfation (13, 17, 18). We have previously described the increased expression of a core 3 disialyl-Lewis^x^ glycan in colorectal tumors, competing with its sulfo sialyl-Lewis^x^ counterpart in normal tissue (19).

Biosynthesis of mucin O-glycans is governed by several glycosyltransferases. It has been shown that during malignant transformation the level of expression of genes encoding glycosyltransferases as well as activities and Golgi localization of these enzymes are modified, leading to less complex structures or novel glycans (16, 20, 21). Overexpression of core 1 β1,3 galactosyltransferase (core 1 synthase) is associated with tumor growth and metastasis of colon cancer cells and with poor survival (22). Downregulation of core 2 β1,6 *N*-acetylglucosaminyltransferase (core 2 synthase) and β1,3 *N*-acetylglucosaminyltransferase 6 (core 3 synthase) may be involved in maintaining the tumor phenotype (15, 21, 23–25).

The changes observed in colorectal cancer at the mucin O-glycosylation level reflect the alterations of gene expression of glycosyltransferases involved in their biosynthesis. Nevertheless, the accumulation of a given structure does not necessarily indicate which glycosyltransferase gene is altered. Indeed, multiple changes can lead to the accumulation of specific structures. For instance, it has been shown that sialyl-Lewis^x^ motifs accumulation can result either from an absence of sulfation of the type 1 chain Galβ1-3GlcNAc allowing its sialylation or from an absence of α2-6 sialylation of the core GalNAc that is replaced by the synthesis of the type 2 chain Galβ1-4GlcNAc further sialylated to form sialyl Lewis^x^ motifs (26).

However, despite all these studies, many of the efforts focused on differential glycosylation of mucins on tumor detection and therapeutics remain investigative. Thus, in this work, we explored the differential expression of mucins and the expression level of the major O-glycans. The objective was to identify differences in mucin glycosylation between CRC of various stages and grades in order to propose new predictive or prognostic biomarkers in colon cancer.

## Results

### Cancer-associated alterations in mucin O-glycosylation are not restricted to the tumor but spread in resection margins

To investigate potential differences in the profile of glycosylation of colon mucins during malignant transformation, colorectal tumor tissues were collected from 10 patients with tumors of different stages and grades (Table 1). Resection margins from the same patients were included in this study as well as five control mucosae from healthy individuals.

**Table 1:** **Characteristics of the tumor samples (and their paired resection margins) used in this study**.

Patient	Age	Gender	Tumor stage	Location	Differentiation	Recurrence
1	46	Female	III	Right	Poor	No
2	55	Male	II	Right	Moderate	No
3	97	Female	II	Transverse	Moderate	Yes
4	69	Male	IV	Right	Moderate	Yes
5	64	Female	II	Transverse	Moderate	No
6	67	Male	II	Right	Well	No
7	82	Male	III	Left	Moderate	Yes
8	80	Female	IV	Right	Poor	No follow-up
9	62	Male	III	Sigmoid	Moderate	No
10	67	Male	IV	Left	Mucinous	No

Purified mucins were analyzed for their glycan compositions and sequences. Oligosaccharides were released by base/borohydride treatment followed by desalting. The pool of oligosaccharide alditols obtained from each sample was thus permethylated before analysis by matrix-assisted laser desorption ionization–time of flight (MALDI-TOF) tandem MS and nano ESI Q-TOF MS/MS as previously published (27–29). The relative abundance of oligosaccharides was calculated based on integration of peaks on MS spectra.

**Figure 1:**
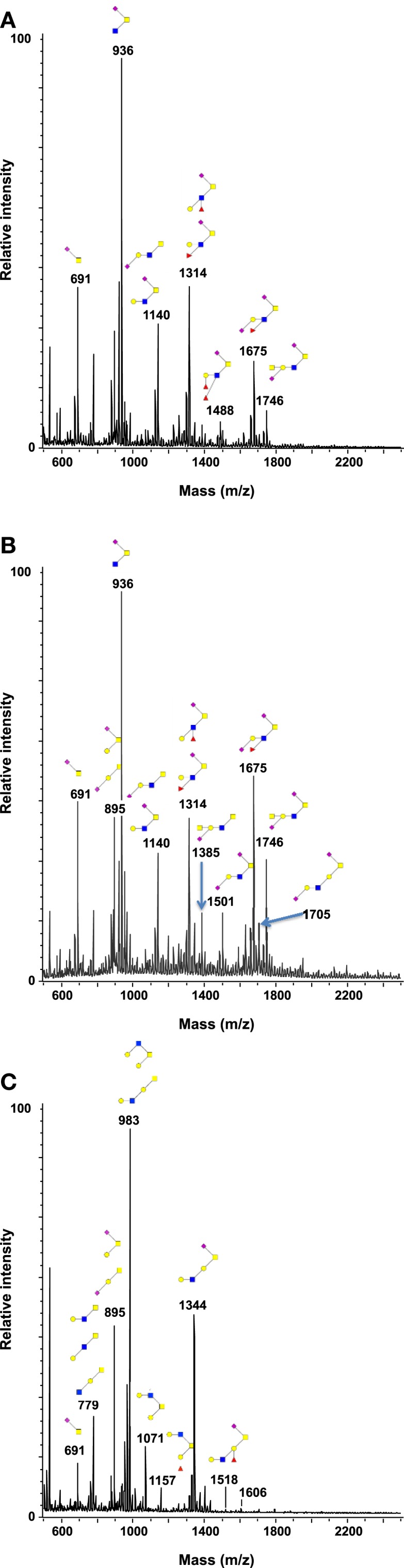
**Structural characterization of human colorectal mucins O-glycans**. MALDI-MS spectra, acquired in the positive ion mode [M + Na]^+^ (or [M + 2Na-H]^+^ for sulfated species), of human intestinal permethylated O-glycans from mucins purified from **(A)** healthy colon controls; **(B)** resection margins; **(C)** paired tumoral tissue. Monosaccharide symbols according to the Consortium for Functional Glycomics (CFG) nomenclature. Key: fucose (red triangle), GlcNAc (blue square), sialic acid (purple diamond), galactose (yellow circle), GalNAcol (yellow square), and sulfate residue (S).

As shown in Figure 1 and Table 2, more than 60 ions corresponding to O-glycans carried by mucins were identified in this study. The O-glycans were 2–10 residues long and were mostly sialylated. Tumors presented the highest diversity, only one of the structure identified here being absent in these samples whereas 16 of them were absent in healthy controls and 11 in resection margins. Most of the oligosaccharides recovered in healthy mucosae were based on a sialylated core 3 structure [GlcNAcβ1-3(NeuAcα2-6)GalNAcol], as described previously (19, 30). For example, expression of this trisaccharide on MS spectra, corresponding to the ion at *m/z* 936, represented around 30% of total glycans in healthy mucosae. A decreasing gradient of structures based on a core 3 was observed from healthy mucosae to tumoral samples, correlating with an increasing gradient of expression of structures based on a core 1 (Galβ1-3GalNAcol). Core 3-based O-glycans represented 64% of the O-glycans population in healthy mucosae, 56.5% in resection margins and only 27.5% of total O-glycans in tumors. The trisaccharidic core 3 structure detected *via* the ion at *m/z* 936 dropped from 31% in healthy mucosa to 12% in the tumor. On the contrary, core 1 oligosaccharides ranged from 13% in healthy mucosae to 21% in resection margins and 41% in tumors. Levels of expression of core 1 and core 3 between controls and tumors were significantly different (*p*-value of 0.0038 and 0.0062, respectively).

**Table 2:** **Proposed neutral and acidic oligosaccharide structures or sequences identified in human mucins from tumors and resection margins of patients with colorectal cancers and from colonic mucosa of healthy individuals**.

Proposed structures or sequences of oligosaccharides	[M **+** Na]^+^ or [M **+** 2Na-H]^+^ for sulfated species	Tumors	Resection margins	Healthy controls
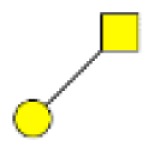	534	5.6 ± 5.7	1.8 ± 2.1	1.0 ± 1.4
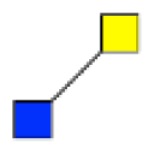	575	1.7 ± 1.5	5.8 ± 6.7	3.8 ± 2.5
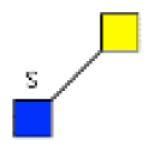	663	0.2 ± 0.4	0.3 ± 0.6	1.0 ± 1.4
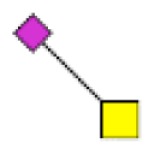	691	7.1 ± 7.1	5.2 ± 5.3	5.8 ± 4.9
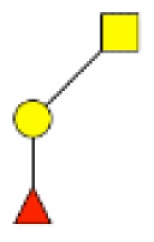	708	0.5 ± 1	0.3 ± 0.6	0.4 ± 0.5
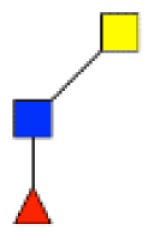	749	0.4 ± 0.8	1.0 ± 2.2	0
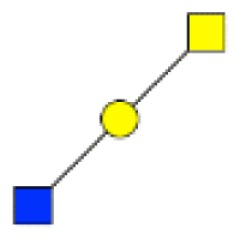 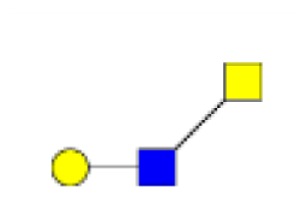 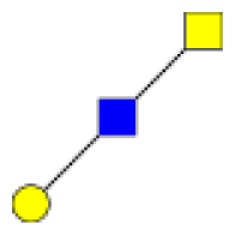	779	4.2 ± 1.5	5.0 ± 3.8	5.6 ± 0.4
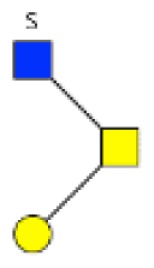	867	0.8 ± 1.1	0.3 ± 0.6	0
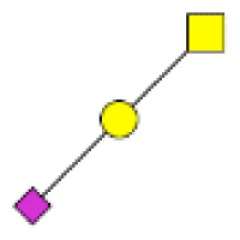 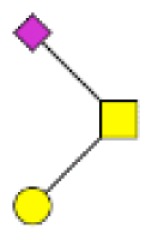	895	10.9 ± 3.6	7.7 ± 4.2	5.7 ± 0.6
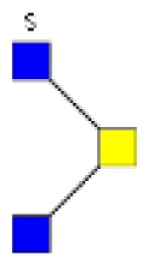	908	0.6 ± 1.0	1.3 ± 1.8	0.8 ± 1.1
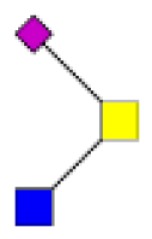	936	12.1 ± 11.2	24.2 ± 10.2	30.8 ± 11.6
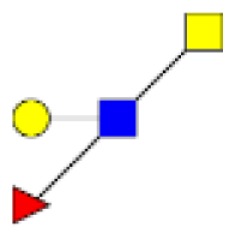	953	1.4 ± 0.6	3.0 ± 1.8	1.9 ± 0.7
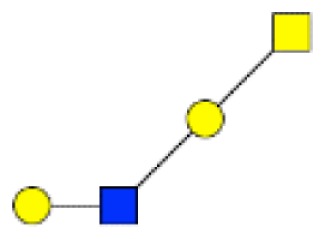	983	7.5 ± 8.3	2.0 ± 0.7	1.6 ± 0.3
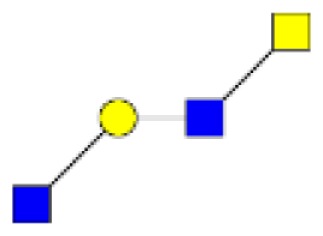	1,024	0.2 ± 0.4	0.4 ± 0.5	0.3 ± 0.5
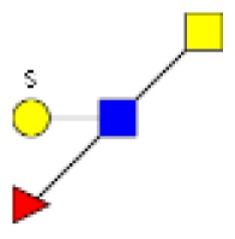	1,041	0.0 ± 0.1	0.3 ± 0.6	0.2 ± 0.2
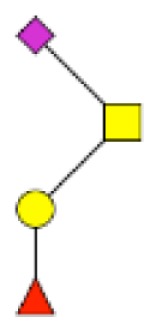	1,069	0.2 ± 0.4	0.2 ± 0.4	0.8 ± 0.1
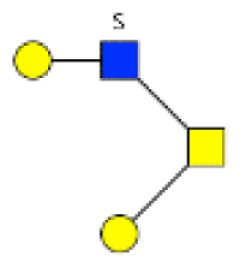	1,071	0.6 ± 1.5	0	0
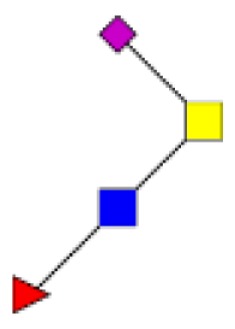	1,110	0.3 ± 0.5	0.7 ± 1.1	0.7
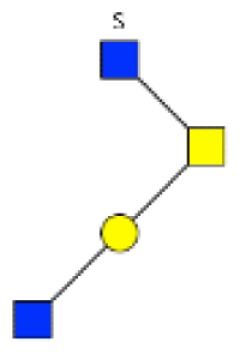	1,112	0.1 ± 0.2	0.2 ± 0.3	0.3 ± 0.4
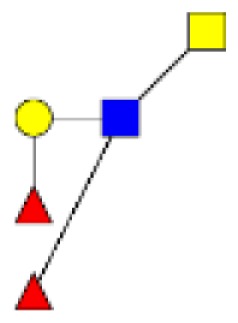	1,127	0	0.3 ± 0.8	0
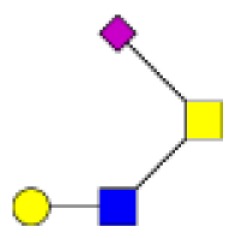 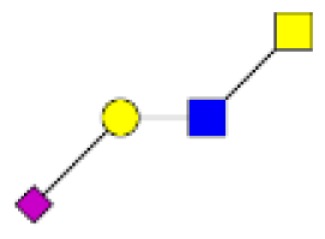	1,140	2.9 ± 2.8	4.4 ± 1.9	7.0
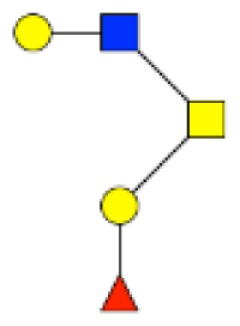 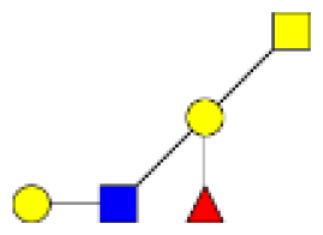	1,157	1.9 ± 1.2	0.4 ± 0.7	0.3 ± 0.4
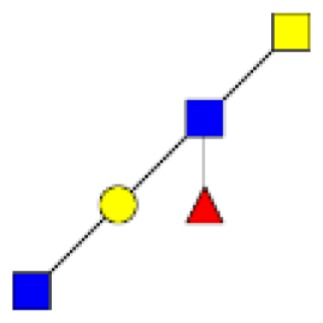	1,198	0.0 ± 0.1	0.4 ± 0.6	0
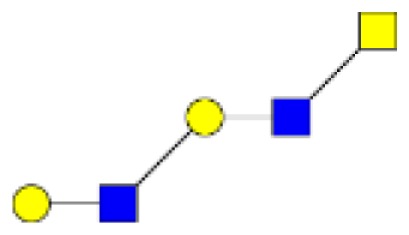	1,228	0.8 ± 0.5	0.5 ± 0.5	0.6 ± 0.2
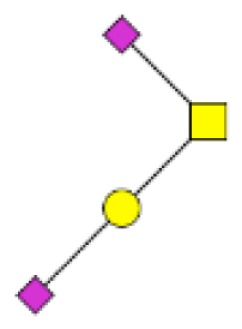	1,256	3.5 ± 1.8	2.7 ± 4.0	1.5 ± 0.4
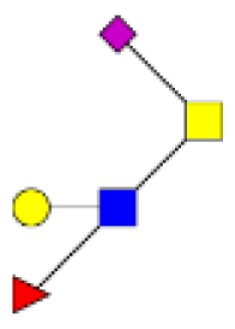 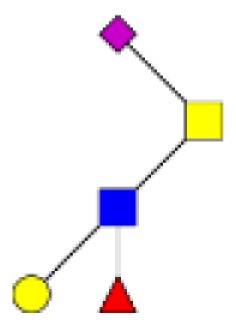	1,314	3.3 ± 2.4	7.0 ± 4.3	8.0 ± 1.4
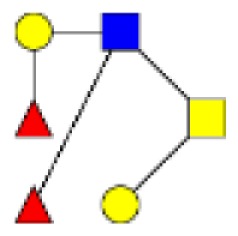	1,331	0.1 ± 0.3	0.1 ± 0.4	0.1 ± 0.2
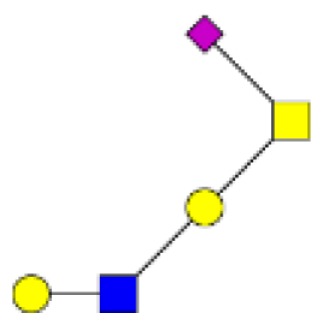	1,344	7.5 ± 5.5	3.3 ± 6.0	1.1 ± 0.4
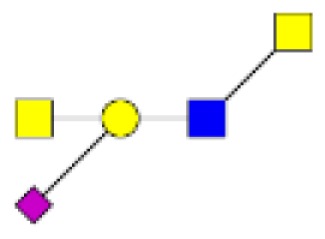	1,385	0.7 ± 1.4	1.9 ± 2.3	0.7 ± 1.0
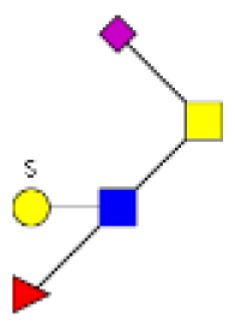	1,402	0.8 ± 0.6	3.2 ± 1.4	7.1 ± 0.3
3 Hex, 2 HexNAc, GalNAcol	1,432	0.9 ± 0.8	0.2 ± 0.2	0.2 ± 0.3
2 Hex, 3 HexNAc, GalNAcol	1,473	0.1 ± 0.2	0.1 ± 0.2	0.2 ± 0.3
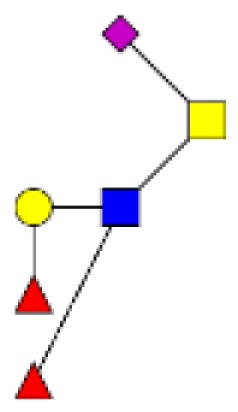	1,488	0.3 ± 0.6	0.8 ± 1.7	0.7 ± 1.0
2 Hex, 2 HexNAc, 1 Fuc, 1 SO3, GalNAcol	1,490	0.2 ± 0.4	0	0
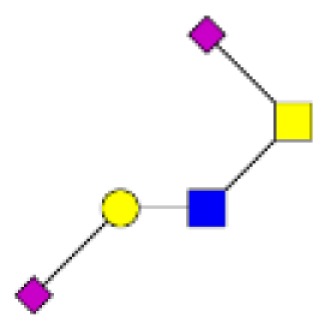	1,501	0.5 ± 0.6	0.6 ± 0.9	1.6 ± 0.7
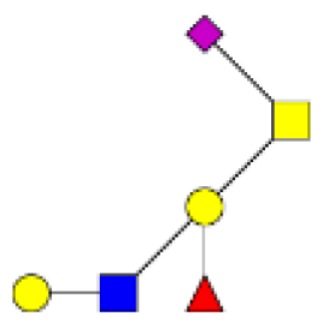	1,518	1.7 ± 1.8	0.4 ± 0.6	0.2 ± 0.4
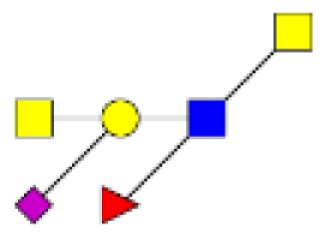	1,559	0.1 ± 0.2	0.1 ± 0.2	0.1 ± 0.2
2 Hex, 2 HexNAc, 2 Fuc, GalNAcol	1,576	0 ± 0.1	0	0.1 ± 0.2
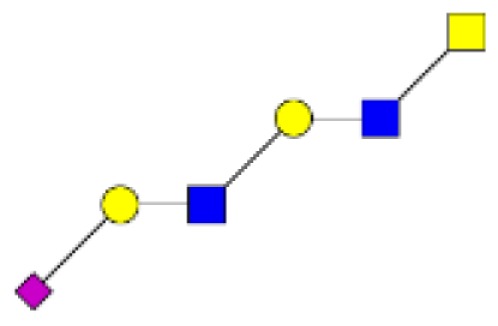	1,589	0.3 ± 0.3	0.3 ± 0.5	0.4 ± 0.1
3 Hex, 2 HexNAc, 1 Fuc, GalNAcol	1,606	0.7 ± 0.8	0	0.1 ± 0.1
2 Hex, 3 HexNAc, 1 Fuc, GalNAcol	1,647	0.6 ± 0.8	0.2 ± 0.4	0.3
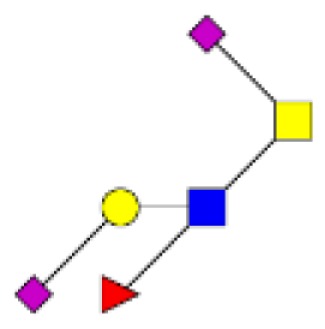	1,675	9.4 ± 1.2	2.5 ± 2.4	3.3 ± 0.7
3 Hex, 3 HexNAc, GalNAcol	1,677	1.0 ± 1.3	0.4 ± 1	0
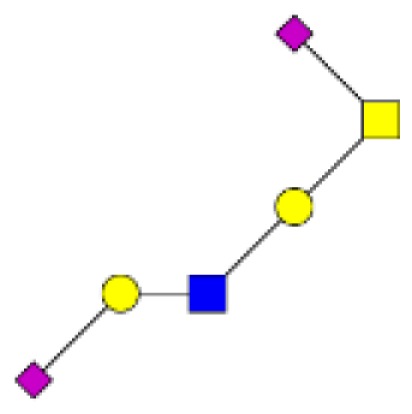	1,705	3.2 ± 3.4	2.5 ± 6.0	0.6 ± 0.1
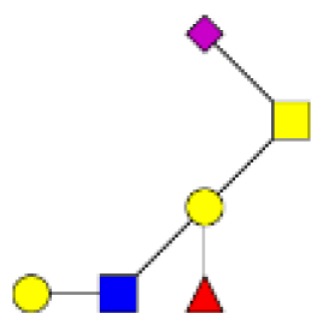	1,718	0.2 ± 0.3	0.1 ± 0.3	0.1 ± 0.1
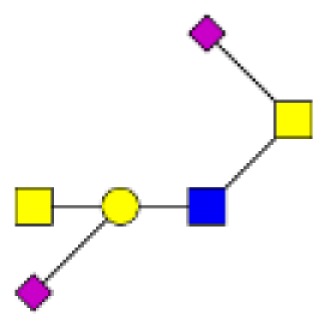	1,746	0.9 ± 2	1.9 ± 2.3	1.1 ± 1.5
2 Hex, 2 HexNAc, 1 Fuc, 1 NeuAc, GalNAcol	1,763	0.2 ± 0.2	0.2 ± 0.3	0.3 ± 0.1
3 Hex, 2 HexNAc, 2 Fuc, GalNAcol	1,780	0.5 ± 0.8	0	0
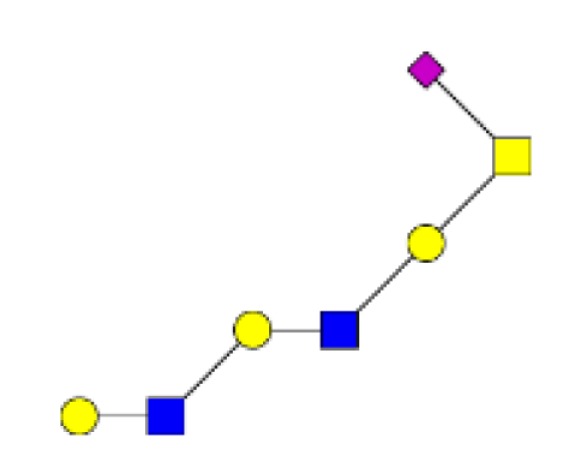	1,793	0.3 ± 0.4	0.2 ± 0.4	0
2 Hex, 3 HexNAc, 1 NeuAc, GalNAcol	1,834	0.1 ± 0.1	0.2 ± 0.3	0.1 ± 0.1
3 Hex, 3 HexNAc, 1 Fuc, GalNAcol	1,851	0.4 ± 0.8	0	0.1 ± 0.1
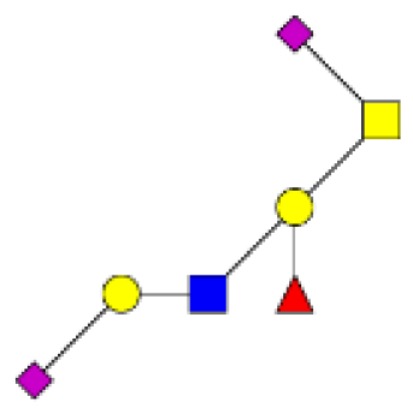	1,879	0.2 ± 0.5	0.3 ± 0.8	0
4 Hex, 3 HexNAc, GalNAcol	1,881	0.4 ± 0.7	0	0
4 Hex, 2 HexNAc, 1 Fuc, 1 SO3, GalNAcol	1,898	0 ± 0.1	0.1 ± 0.2	0
3 Hex, 4 HexNAc, GalNAcol	1,922	0.2 ± 0.5	0.1 ± 0.3	0.1 ± 0.1
2 Hex, 2 HexNAc, 2 Fuc, 1 NeuAc, GalNAcol	1,937	0 ± 0.1	0	0.1 ± 0.1
3 Hex, 3 HexNAc, 1 Fuc, 1 SO3, GalNAcol	1,939	0.1 ± 0.2	0	0
3 Hex, 2 HexNAc, 3 Fuc, GalNAcol	1,954	0.1 ± 0.2	0	0
4 Hex, 2 HexNAc, 1 NeuAc, GalNAcol	1,997	0.1 ± 0.2	0	0
3 Hex, 3 HexNAc, 2 Fuc, GalNAcol	2,025	0.4 ± 0.5	0.1 ± 0.2	0.1 ± 0.1
5 Hex, 3 HexNAc, GalNAcol	2,055	0.6 ± 0.9	0	0

*The relative percentage of each oligosaccharide was calculated based on the integration of peaks on MS spectra. Results are presented as the mean ± SD of percentage of each oligosaccharide for a same condition*.

*Monosaccharide symbols according to the Consortium for Functional Glycomics (CFG) nomenclature. Key: fucose (red triangle), GlcNAc (blue square), sialic acid (purple diamond), galactose (yellow circle), GalNAcol (yellow square), and sulfate residue (S)*.

**Figure 2:**
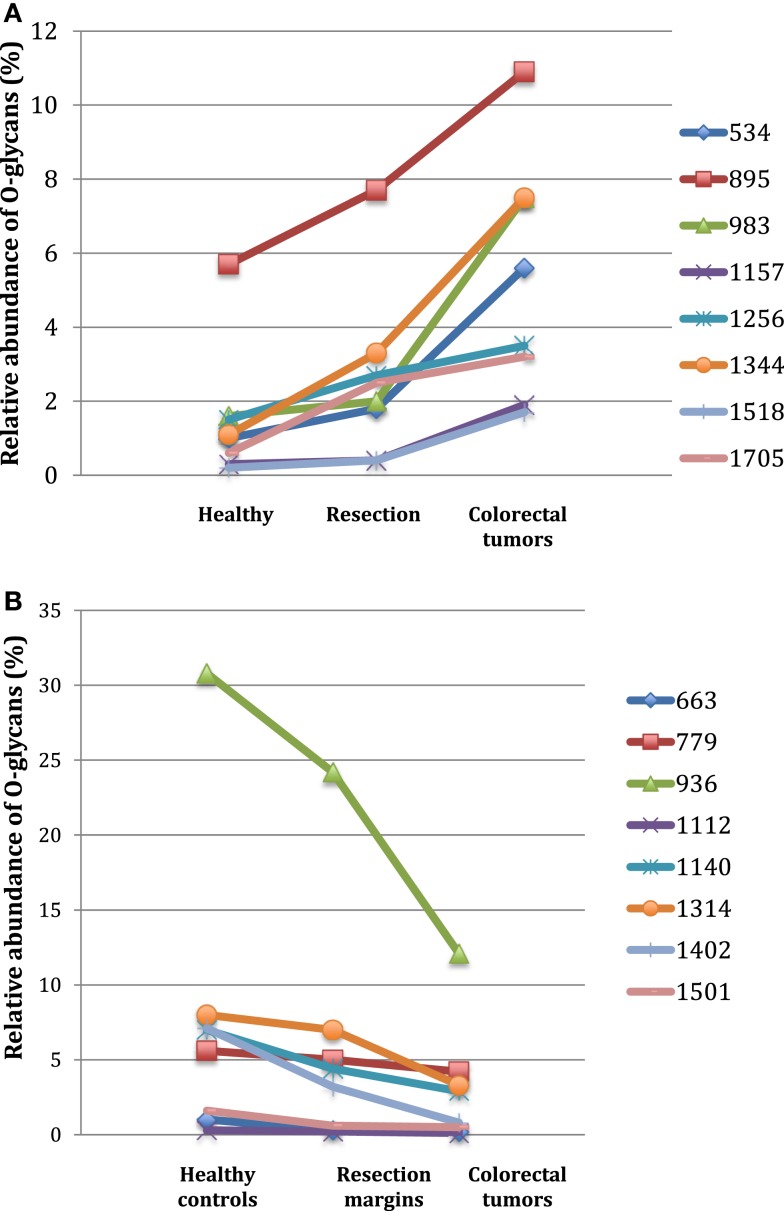
**Gradients in the level of expression of mucin O-glycans from healthy controls colonic tissues (*n* **=** 5) to resection margins (*n* **=** 10) and paired tumors of patients (*n* **=** 10) with colorectal cancers**. The mean is represented for all measurements. **(A)** CRC-specific increase in the level of expression of O-glycans corresponding to ions at *m/z* 534, 895, 983, 1,157, 1,256, 1,344, 1,518, and 1,705. **(B)** CRC-specific decrease in the level of expression of O-glycans corresponding to ions at *m/z* 663, 779, 936, 1,112, 1,140, 1,314, and 1,402.

Among the 60 O-glycans identified, around one-third followed either a decreasing gradient of expression or an increasing gradient from healthy controls to resection margins and tumors (Figure 2). A decrease in the level of expression of sialylated core 3 structures at *m/z* 936, 1,140, and 1,314 was observed in tumors compared to resection margins and controls, the relative percentage of these three O-glycans was 18, 36, and 46% of total glycans, respectively. An increased level of expression of sialylated core 1 structures seemed to correlate with the decrease of core 3 glycans, the relative percentage of ions at *m/z* 895, 1,344, 1,518, and 1,793 corresponded to 7% in controls, 12% in resection margins, and 21% in tumors.

As previously described (19), an increase in the disialyl-Lewis^x^ antigen at *m/z* 1,675 was observed in the tumor samples (9.4% of total O-glycans) compared to resection margins (2.5%) and controls (3.3%). Its 6-sulfo sialyl-Lewis^x^ counterpart decreased in tumors (0.8% of total glycans) compared to resection margins (3.2%) and controls (7.1%). Other significant differences between tumors and controls concerned the important increase in the level of expression of ions at *m/z* 534 (from 1 in controls to 5.6% in tumors); 983 (from 1.6 to 7.5%); 1,344 (from 1.1 to 7.5%); and 1,705 (from 0.6 to 3.2%). Interestingly, all these core 1-based glycans seem to follow the same biosynthetic pathway sequence: the T antigen at *m/z* 534 is first elongated by a GlcNAc residue to give the ion at *m/z* 779. A galactose residue is then linked to the GlcNAc residue to give the ion at *m/z* 983, which will further be substituted by one sialic acid residue (to give the ion at *m/z* 1344) or two sialic acid residues, giving the ion at *m/z* 1705.

The total sialylation level of mucin O-glycans did not significantly vary between tumors, resection margins and healthy controls and represented 67, 71, and 78% of total O-glycans, respectively. A decrease in the level of sulfation was observed in tumors (only 3.4% of total oligosaccharides) compared to resection margins (5.7%) and controls (9.4%).

Generally, the levels observed for the resection margins are intermediate between the situation in healthy mucosa and what is observed in tumors. It has been shown that inflammation can affect the O-glycosylation profile, for instance, in the case of acute ulcerative colitis (31). Micro-inflammation could therefore explain the observed glycosylation pattern of resection margins. SD values (Table 2) indicated that levels of expression of oligosaccharides from tumors showed more heterogeneity and inter-individual variations than those from healthy controls. This result may be explained by the fact that tumoral samples presented different anatomo-pathological features such as degree of histological differentiation or tumoral stage.

### Comparison of the repertoire of glycosylation of mucin O-glycans according to the tumor stage

Colorectal cancers are most commonly classified using the TNM staging system. This system takes into account three key factors that are the invasiveness of the tumor (T), lymph nodes (N), and metastasis (M). Once the T, N, and M scores have been assigned (each of three factors are assigned either with a number 0–4 for T or 0–1 for N and M), an overall stage is determined, and thus treatment options may be proposed and explored. In stage 0, the cancer has not grown beyond the inner layer of the colon or rectum. In stage I, the cancer has grown into the submucosa and muscularis propria whereas in stage II the cancer has grown through all the layers of the colon or rectum with or without the perforation of visceral peritoneum. In stage IIc, the cancer has also grown into nearby organs or tissues, in contrast to stage IIb. Stage III is divided into three categories, depending on the extent to which the cancer has spread, and how many lymph nodes have been affected. Stage IV is the most advanced stage of colorectal cancers; it has metastasized to distant sites such as the liver or the lung. The cancer may or may not have grown through the wall of the colon or rectum, and lymph nodes may or may not have been affected.

**Figure 3:**
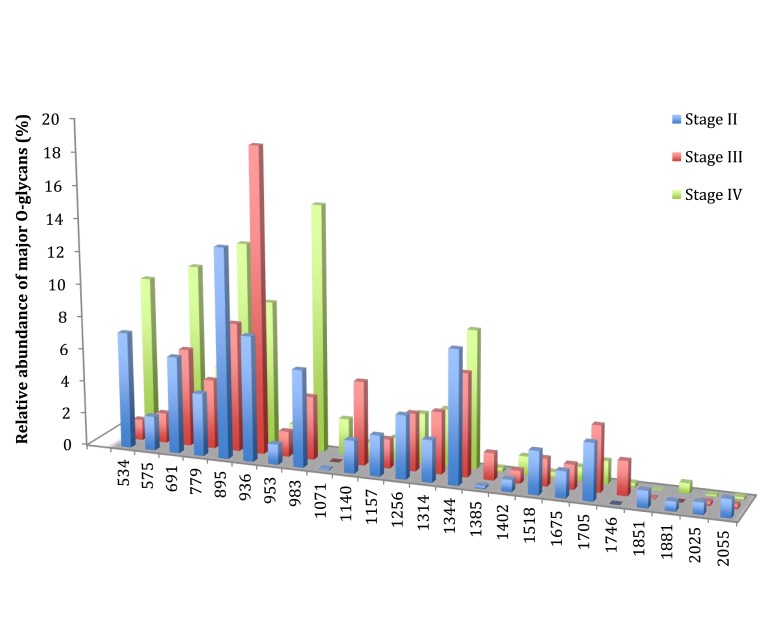
**Comparison of the relative abundance of major mucin O-glycans purified from tumors of stage II (*n* **=** 4), stage III (*n* **=** 3), or stage IV (*n* **=** 3)**. A low increase in the level of expression of ions at *m/z* 534, 691, and 983 was observed in stage IV colorectal tumors, compared to stages II and III.

In this study, four patients presented a cancer of stage II, three were of stage III, and three of stage IV. Compared to stages II and III, stage IV was characterized by a low increase in the level of expression of three ions: the ion at *m/z* 534, corresponding to the T antigen represented 7.1% of total O-glycans in stage II to 1.2% in stage III and 9.7% in stage IV (Figure 3). The ion at *m/z* 691, corresponding to the sialyl Tn antigen increased from 5.9% of total O-glycans in stage II, 6% in stage III, and 10.7% in stage IV. The level of expression of the last ion at *m/z* 983, corresponding to a core 1 tetrasaccharide was 6% of total glycans in stage II, 3.9% in stage III, and 15.1% in stage IV. However, no direct link between carcinogenesis stages and O-glycans structure profile could be drawn from these results.

### Tumors with recurrence can be distinguished from tumors without recurrence

**Figure 4:**
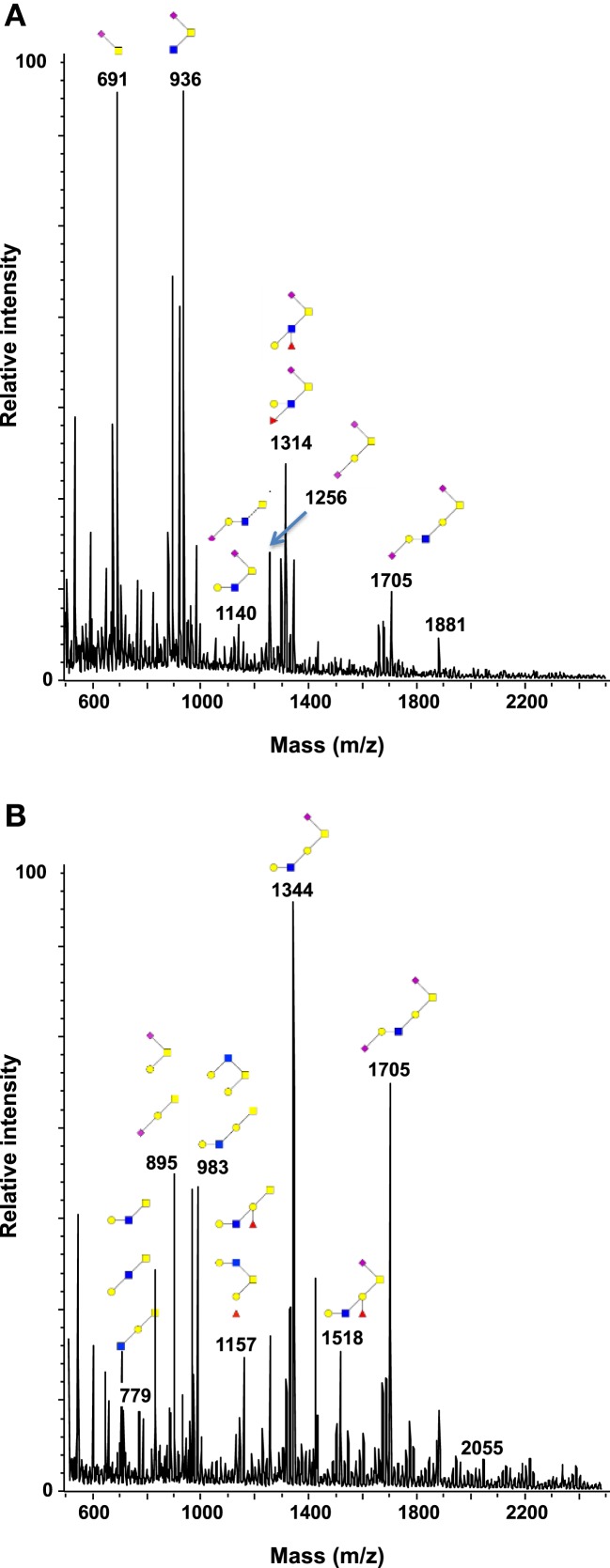
**Comparison of the glycosylation pattern of mucins purified from colorectal tumors with or without recurrence**. MALDI-MS spectra, acquired in the positive ion mode [M + Na]^+^ (or [M + 2Na-H]^+^ for sulfated species), of human intestinal permethylated O-glycans from mucins purified from **(A)** tumors with recurrence; **(B)** tumors without recurrence. Monosaccharide symbols according to the Consortium for Functional Glycomics (CFG) nomenclature. Key: fucose (red triangle), GlcNAc (blue square), sialic acid (purple diamond), galactose (yellow circle), GalNAcol (yellow square), and sulfate residue (S).

**Figure 5:**
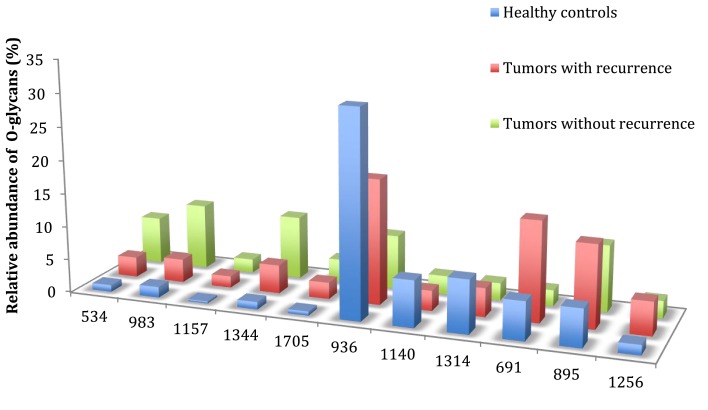
**Comparison of the relative abundance of major mucin O-glycans between healthy controls (*n* **=** 5), tumors with recurrence (*n* **=** 3), and tumors without recurrence (*n* **=** 6)**. The mean is represented for all measurements. A decrease in the level of expression of core 3-based glycans from healthy controls to tumors without recurrence is seen with the ions at *m/z* 936, 1,140, and 1,314 and an increase in the core 1-based glycans is represented by the ions at *m/z* 534, 983, 1,157, and 1,344. Tumors with recurrence expressed higher levels of sialyl Tn (ion at *m/z* 691), sialyl T (ion at *m/z* 895), and disialyl T antigens (ion at *m/z* 1,256), compared to healthy controls and tumors without recurrence.

In this study, all patients underwent curative resection for colorectal cancers. The profiles of mucin O-glycans released from tumors with recurrence and from tumors without recurrence were compared and confronted to the dataset from healthy controls. Figures 4 and 5 highlight differences between samples in the level of expression of oligosaccharides. Three kinds of differences could be identified: ions at *m/z* 534, 983, 1,157, 1,344, and 1,705, all corresponding to core 1 glycans, showed an increasing gradient of expression from healthy controls to tumors without recurrence. Tumors with recurrence presented an intermediate level of expression of these glycans.

On the contrary, ions at *m/z* 936, 1,140, and 1,314, all corresponding to sialylated core 3 glycans, showed a decreasing gradient of expression from controls to tumors without recurrence, with again an intermediate state for tumors with recurrences.

The last kind of differences concerned sialyl Tn, sialyl T, and disialyl T antigens at *m/z* 691, 895, and 1,256, respectively. Each of these three antigens was more expressed in tumors with recurrence than in tumors without recurrence and healthy controls. The levels of expression of sialyl Tn antigen between the two types of tumors were significantly different (*p* = 0.012).

## Discussion

Colorectal cancer is the third most commonly diagnosed cancer in males and the second in females, with over 1.2 million new cases and around 600,000 deaths estimated to have occurred worldwide in 2008 (32). Surgical resection is the primary treatment modality for stages I–III CRC, and the most powerful tool for assessing prognosis following curative surgery remains pathologic analysis of the resected specimen. Currently, the parameters that determine pathologic stage are the strongest predictors of postoperative outcome. An intensive surveillance for patients with resected cancer is highly recommended, and is based on a clinical encounter with a physician every 3–6 months for the first 3 years and serial measurement of carcinoembryonic antigen (CEA) at each follow-up visit. However, approximately one in three people who develop CRC dies of this disease and more than 40% will have a disease recurrence, despite development of screening tests and surveillance for CRC in patients with colon polyps and/or family history and despite progress in surgical resection of tumors, adjuvant chemotherapy, and postoperative surveillance. Therefore, the challenge is to identify new cancer markers for prediction of the risk of recurrence after potentially curative resection.

Aberrant expression and glycosylation of mucins is a common feature of all adenocarcinomas (14, 33, 34). In colorectal cancer, the differences from normal mucins have been described both at the gene and post-translational levels. The synthesis of MUC2, the main intestinal secreted mucins, has been shown to be suppressed in colorectal cancer and its metastases (35). Distribution of MUC2 within goblet cells changes in adenomas compared to healthy mucosa, with, for example, an increased presence in the cytoplasm and Golgi apparatus and a decreased localization in the vesicle (36). A *de novo* expression of MUC5AC, a gastric foveolar mucin has been described in mucinous adenocarcinomas as well as in tumors exhibiting microsatellite instability (37–39). MUC5AC and MUC6 were also found in a high proportion of villous and tubulovillous adenomas but not in normal colonic biopsies (40). No significant association between high mucin content in colorectal cancer and overall survival or celerity of disease progression has yet been demonstrated (41).

Alterations of mucin glycosylation in colorectal cancer include reduced number and length of carbohydrate side chains on apomucins, deletion of normally expressed antigens, *de novo* appearance of novel antigens, and expression of blood group-incompatible antigens (20, 33, 34, 42). Although mucin O-glycans have long been implicated in colorectal carcinogenesis, none of them have been explored as potential marker of recurrence. In this study, we compared the O-glycosylation profile of mucins from tumors with or without recurrence and have observed important differences in the level of expression of O-glycans between these two types of samples.

First of all, the level of expression of sialyl Tn antigens at *m/z* 691 is significantly higher in tumors with recurrence, compared to tumors without recurrence. This result is in agreement with previous studies demonstrating that overexpression of Tn and sialyl Tn antigens are characteristics of more advanced and poorly differentiated colon cancers (8, 43). In a recent study conducted by Chik et al., overexpression of sialyl Tn antigens was correlated with upregulation of alpha 2,6 sialyltransferase gene (*ST6GALNAC1*) and a decrease in core 1 synthase gene (*C1GALT1*) in the mucinous colorectal cancer cell line LS174T (44). Interestingly, our analysis of mucin glycosylation of tumors with recurrence demonstrated a higher proportion of core 3 O-glycans, and a weaker expression of core 1 O-glycans, compared to tumors without recurrence. These data seem to indicate a less active or expressed core 1 synthase in tumors with recurrence, compared to tumors without recurrence. This result could, at least partially, explain the higher expression of sialyl Tn antigens in tumors with recurrence.

A generally higher level of expression of sialyl T and disialyl T antigens was also observed in tumors with recurrence, compared to tumors without recurrence, although these differences were not significant. Sialylation of the T antigen is governed by action of ST6GalNAc-II, and an overexpression of its mRNA level has been correlated to poor patient survival in CRC with lymph node metastases (45). The incomplete elongation of O-glycans resulting in the abundance of these sialylated truncated structures in tumors with recurrence may have different origins. Epigenetic silencing of glycosyltransferase genes necessary for the synthesis of more complex O-glycan core structures may, for instance, be involved, for some of related enzymatic activities are known to be significantly decreased in cancers (20, 46) Down regulation of the genes encoding core 1 enzyme and/or its chaperon Cosmc may also lead to the appearance of Tn and sialyl Tn antigens (47, 48). Hypoxia-induced transcription modification of several glycosyltransferase genes may also be involved in advanced tumoral stage (49).

In this work, we compared the pattern of glycosylation of mucins from three tumors with recurrence and six tumors without recurrence. Even if numbers of cases were relatively small, the most striking feature of O-glycans from tumors with recurrence, compared to tumors without recurrence, concerned the balance between the level of expression of core 1- and core 3-based glycans. In tumors without recurrence, a strong decrease in core 3-based O-glycans, correlated to a high increase in core 1-based O-glycans, was observed. By contrast, in tumors with recurrence, the level of expression of core 3-based oligosaccharides remains important and only a slight increase in the proportion of core 1 O-glycans can be seen. Compared to healthy controls, the profile of glycosylation of mucins from tumors with recurrence is less altered than the one of tumors without recurrence. In other words, mucin O-glycosylation from tumors with recurrence looks at least partially like the one of healthy controls. The high proportion of core 3 O-glycans in tumors with recurrence may contribute to mimic normal physiological conditions, rendering the immune defense system less efficient to fight the tumor cells.

In this study, we also compared the profile of glycosylation of mucins between tumors, resection margins, and healthy controls. No significant differences in the level of expression of Sda/Cad antigens were observed between these three types of samples. Previous studies have reported a loss of Sda synthase and Sda antigens in colorectal cancers (50–52). Our precedent study on glycosylation of MUC2 in three human colorectal carcinomas allowed us to demonstrate that the disialylated glycans with Sda/Cad epitope (corresponding to the ion at *m/z* 1746) were both recovered in normal and cancerous tissues whereas a decrease in the monosialylated glycans with Sda/Cad epitopes was found (19). This study confirms that mucins from colorectal carcinomas still express Sda/Cad antigens. One explanation for the absence of differences in the level of expression of these antigens between healthy controls and tumors may come from the origin of samples. Indeed, it has been demonstrated that Sda/Cad antigens are mainly expressed in the descending colon (29, 53) and results are presented here as the mean of percentage of each oligosaccharide for all samples from the same condition. Taken individually, results showed great differences in the level of expression of these antigens (data not shown).

Comparison of glycosylation between tumors, resection margins, and healthy controls indicate the presence of gradients of expression of major O-glycans: core 3 O-glycans decreased from healthy controls to tumors, and an increasing gradient of core 1 O-glycans was recovered. This result probably arises from alterations in the level of expression of genes encoding glycosyltransferases or in the activities of such enzymes. Previous studies have demonstrated, by quantitative real time RT-PCR, an overexpression of *C1GALT1* in tumors of patients with colorectal cancer, promoting the invasive behavior of tumoral cells (22, 54). Other studies, using quantitative real time PCR and immunohistochemistry have shown a downregulation of core 3 synthase in carcinomas (24).

Comparison of the glycosylation patterns of mucins from resection margins and healthy controls shows that alterations of mucin O-glycans are not only recovered in tumors but also in resection margins. This suggests that, even if margins of resection are macroscopically and anatomically safe, molecular alterations are nevertheless present and may contribute to the risk of recurrence for certain patients.

In this study, no correlation was established between mucin O-glycans and the stage of colorectal cancers. Variations in the level of expression of certain glycans have been observed, such as a higher expression of ions at *m/z* 534 (T antigen), 691 (sialyl Tn antigen), and 983 in stage IV. However, none of these alterations were significant. Even if numbers of samples in each stage was relatively low, one hypothesis may be that modifications of mucin glycosylation are an early step during colorectal carcinogenesis.

In conclusion, our work demonstrated for the first time a possible correlation between mucin O-glycosylation patterns in the resected tumor of patients and the risk of recurrence. These findings might prove valuable as prognostic tools orienting the cure to be given to patients after resection.

## Materials and Methods

### Human samples and mucin preparation

Fresh tissue samples from 10 individuals with colorectal carcinoma were taken at surgery. Specimens were obtained from the tumor as well as from resection margins. A summary of localization of tissue sampling, tumor stage, grade of differentiation, age, and sex of the donors is given in Table 1. Colorectal tissues from five healthy individuals were also included in this study. These samples were healthy resection margins of colonic tissues arising from patients with diverticulosis. The use of human tissues for this study was approved by the local hospital ethics committee and French Ministry of Higher Education and Research (DC-2008-242). All subjects gave written informed consent in accordance with the Declaration of Helsinki.

Samples were frozen and stored at −20°C until used. A pathologist at the Saint Vincent Hospital of Lille carried out the CRC diagnosis of tumors. All samples were obtained with informed consent of the patients.

### Isolation and purification of mucins from colorectal tissues

Mucins were solubilized in 4 M guanidine chloride solution containing 10 mM dithiothreitol, 5 mM ethylenediaminetetraacetic acid, 10 mM benzamidine, 5 mM *N*-ethylmaleimide, 0.1 mg/mL soy bean trypsin inhibitor, and 1 mM phenylmethanesulfonyl fluoride.

Cesium chloride was added to an initial density of 1.4 g/mL and mucins were purified by isopycnic density-gradient centrifugation (Beckman Coulter LE80K ultracentrifuge; 70.1 Ti rotor, 417 600 g at 15°C for 72 h). Fractions of 1 mL were collected from the bottom of the tube and analyzed for periodic acid-Schiff (PAS) reactivity and density. The mucin-containing fractions were pooled, dialyzed against water, and lyophilized.

### Release of oligosaccharides from mucin by alkaline borohydride treatment

The intestinal mucins were submitted to β-elimination under reductive conditions (0.1 M KOH, 1 M KBH4 for 24 h at 45°C). The mixture of oligosaccharides alditols was purified by size exclusion chromatography on a column of Bio-Gel P2 (85 cm × 2 cm ID, 400 mesh, Bio-Rad, Richmond, CA, USA) equilibrated and eluted with water (10 mL/h) at room temperature. The oligosaccharide fractions, detected by UV absorption at 206 nm, were pooled for structural analysis.

### Permethylation and mucin glycosylation analysis by MALDI-TOF mass spectrometry

Permethylation of the mixture of oligosaccharide alditols was carried out with the sodium hydroxide procedure described by Ciucanu and Kerek (55). After derivatization, the reaction products were dissolved in 200 μL of methanol and further purified on a C18 Sep-Pak column (Waters, Milford, MA, USA). Permethylated oligosaccharides were analyzed by MALDI-TOF MS in positive ion reflective mode as [M + Na]^+^. Quantification through the relative percentage of each oligosaccharide was calculated based on integration of peaks on MS spectra.

### Electrospray mass spectrometry (nano-ESI-MS/MS)

All analyses were performed on a Q-STAR Pulsar Q-TOF mass spectrometer (Applied Biosystems/MDS Sciex, Toronto, ON, Canada) fitted with a nano-electrospray ion source (Protana, Odense, Denmark). Oligosaccharides dissolved in water (60 pmol/μL) were acidified by addition of an equal volume of methanol/0.1% formic acid and sprayed from gold-coated “medium length” borosilicate capillaries (Protana). A potential of −800 V was applied to the capillary tip and the focusing potential was set at −100 V, the declustering potential varying between −60 and −110 V. For the recording of conventional mass spectra, TOF data were acquired by accumulation of 10 multiple channel acquisition scans over mass ranges of *m/z* 400–2,000. In the collision-induced dissociation (CID) tandem MS analyses, multiple charged ions were fragmented using nitrogen as collision gas (5.3 × 10−5 Torr), the collision energy varying between −40 and −90 eV to obtain optimal fragmentation. The CID spectra were recorded on the orthogonal TOF analyzer over a range of *m/z* 80–2,000. Data acquisition was optimized to supply the highest possible resolution and the best signal-to-noise ratio even in the case of low-abundance signals. Typically, the full width at half maximum was 7,000 in the measured mass ranges. External calibration was performed prior to each measure using a 4-pmol/μL solution of taurocholic acid in acetonitrile/water (50:50, v/v) containing 2 mM of ammonium acetate.

### Statistical analysis

Student’s *t*-test was used for statistical analysis; *P*-values of < 0.05 were considered statistically significant.

## Conflict of Interest Statement

The authors declare that the research was conducted in the absence of any commercial or financial relationships that could be construed as a potential conflict of interest.

## Abbreviations

CID, collision-induced-dissociation; CRC, colorectal cancer; CsCl, cesium chloride; PAS, periodic acid-Schiff
